# Low Basal CB2R in Dopamine Neurons and Microglia Influences Cannabinoid Tetrad Effects

**DOI:** 10.3390/ijms21249763

**Published:** 2020-12-21

**Authors:** Qing-Rong Liu, Ana Canseco-Alba, Ying Liang, Hiroki Ishiguro, Emmanuel S. Onaivi

**Affiliations:** 1 Laboratory of Clinical Investigation, National Institute on Aging, Baltimore, MD 21224, USA; 2 Department of Biology, William Paterson University, Wayne, NJ 07470, USA; cansecoana@yahoo.com.mx; 3 College of Food Science and Engineering, Central South University of Forestry and Technology, Changsha 410004, Hunan, China; liangying498@163.com; 4 Department of Neuropsychiatry, Graduate School of Medical Science, University of Yamanashi, Chuo, Yamanashi 409-3898, Japan; hishiguro@yamanashi.ac.jp

**Keywords:** cannabinoids, Cre-loxP system, in situ hybridization, CB1 receptor, CB2 receptor, hepatocytes, neurons, microglia, tetrad effects

## Abstract

There are two well-characterized cannabinoid receptors (CB1R and CB2R and other candidates): the central nervous system (CNS) enriched CB1R and peripheral tissue enriched CB2R with a wide dynamic range of expression levels in different cell types of human tissues. Hepatocytes and neurons express low baseline CB1R and CB2R, respectively, and their cell-type-specific functions are not well defined. Here we report inducible expression of CB1R in the liver by high-fat and high sugar diet and CB2R in cortical neurons by methamphetamine. While there is less controversy about hepatocyte CB1R, the presence of functional neuronal CB2R is still debated to date. We found that neuron CB2R basal expression was higher than that of hepatocyte CB1R by measuring mRNA levels of specific isoform CB2A in neurons isolated by fluorescence-activated cell sorting (FACS) and CB1A in hepatocytes isolated by collagenase perfusion of liver. For in vivo studies, we generated hepatocyte, dopaminergic neuron, and microglia-specific conditional knockout mice (Abl-Cnr1^Δ^, Dat-Cnr2^Δ^, and Cx3cr1-Cnr2^Δ^) of CB1R and CB2R by crossing Cnr1^f/f^ and Cnr2^f/f^ strains to Abl-Cre, Dat-Cre, and Cx3cr1-Cre deleter mouse strains, respectively. Our data reveals that neuron and microglia CB2Rs are involved in the “tetrad” effects of the mixed agonist WIN 55212-2, CB1R selective agonist arachidonyl-2′-chloroethylamide (ACEA), and CB2R selective agonist JWH133. Dat-Cnr2^Δ^ and Cx3cr1-Cnr2^Δ^ mice showed genotypic differences in hypomobility, hypothermia, analgesia, and catalepsy induced by the synthetic cannabinoids. Alcohol conditioned place preference was abolished in DAT-Cnr2^Δ^ mice and remained intact in Cx3cr1-Cnr2^Δ^ mice in comparison to WT mice. These Cre-loxP recombinant mouse lines provide unique approaches in cannabinoid research for dissecting the complex endocannabinoid system that is implicated in many chronic disorders.

## 1. Introduction

The classical tetrad effects of marihuana are hypomobility, hypothermia, analgesia, and catalepsy in rodents, and cannabinoid receptor 1 (CB1R) and 2 (CB2R) expressed in various tissues and cell types are the main receptors involved [1,2]. Mouse CB1R expression in the brain is several thousand-fold higher than that of the liver [3], and mouse CB2R expression in the spleen is several hundred-fold higher than that of the brain [4]. CB1R and CB2R were previously called central and peripheral cannabinoid receptors because Northern blot and conventional ISH methods only detected CB1R in brain regions and CB2R in peripheral tissues [5]. Subsequent research demonstrated functional CB1R presence in peripheral tissues [6] and functional neuronal and glial CB2R in brain regions [7]. While there is less controversy of peripheral expression of CB1R, the functional neuronal expression of CB2R has been controversial [8] due to issues of CB2R antibody nonspecificity [9] and because neuron CB2R could not be detected by traditional methods of RT–PCR fragment amplification [10], radio-labeled ligand binding [11], and transgenic green fluorescence protein (GFP) reporter mice [12,13]. However, work from other researchers has been published in recent years showing neuronal CB2R expression in models of CNS functions and dysfunction [14,15,16,17,18].

New molecular techniques and various transgenic approaches are being used to explore the distribution and identify the involvement of elements of the endocannabinoid system (ECS) in the brain and peripheral function and dysfunction [19]. CB1R germline knockout (gKO) mice show phenotypes of weight loss, shortened life span, hypoactivity, and hypoalgesia [20], while CB2R gKO mice show phenotypes of reduced immunomodulatory activity [21] and obesity [22,23]. Otherwise, they appear healthy and fertile. The limitations of gKO are the gene developmental compensations that have confounding effects on the mutant mice. In addition, the CBR gKO mouse models with incomplete deletion of *Cnr1* and/or *Cnr2* genes may retain residue CBR mRNA and protein activities. Zimmer CB1R gKO retains the splicing acceptor site of the coding exon [20], and Buckley CB2R gKO mice is a partial ORF deletion of the C-terminal region [21], and DeltaGen CB2R gKO mice is a partial ORF deletion of the N-terminal region [9].

The first Cnr1^f/f^ mouse strain was generated in Dr. Lutz’s lab [24] by crossing transgenic Cnr1-floxed-Neo-FRT mice with ubiquitously expressed flippase [25] FLP-deleter mice [24,26] to delete the Neo selective marker. Subsequent cross of Cnr1^f/f^ mice with cell-type-specific Cre-deleter mice [27] in several labs produced deletion of *Cnr1* gene in principal neurons [24], glutamatergic or GABAergic neurons [28,29], noradrenergic neurons [30], dorsal root ganglia [31], astrocytes [32,33], adipocytes [34], podocytes [35], keratinocytes [36], and hepatocytes [37] that revealed CB1R cell-type-specific functions in extinguishing fear, epilepsy, osteoporosis, analgesia, learning and memory, obesity, diabetic nephropathy, inflammation, and hepatic steatosis, respectively.

However, CB2R cell-type-specific functional studies lagged behind those of CB1R and are only reported in recent years [2,38,39,40]. CB2R is known to be enriched in blood leukocytes such as different B and T cells, monocytes, macrophages, natural killer cells, neutrophils, eosinophils, basophils, mast cells, [41,42] and plays important roles in immunomodulatory and anti-inflammation resolution by regulating cytokine release, apoptosis, autophagy, induction of T helper cells, suppression of macrophage infiltration, and lymphocyte proliferation [43,44]. Certainly, neuron-specific CB2R cKO mice are invaluable tools in resolving the CB2R controversy that was referred to as a “sphinx” puzzle or CB2R with an identity crisis [8].

With the goal of determining the relative cell-type-specific expression patterns and contribution of cannabinoid receptor subtypes in brain and liver hepatocytes, we report the generation of Cnr1^f/f^ and Cnr2^f/f^ mice to create hepatocyte, dopaminergic neuron, and microglia conditional knock out mice. We found that neuronal CB2R was higher than hepatocyte CB1R expression. While CB2R was detectable in dopaminergic neurons in the VTA [2], we could not detect CB1R in lateral thalamus glutamatergic neurons and in lateral ventral tegmental area (VTA) dopaminergic neurons, using ultrasensitive RNAscope in situ hybridization technique. Using neuronal and microglia conditional CB2R knockout mouse models, we provide evidence of low basal and inducible expression of brain CB2R are involved in alcohol consumption [2] and in the “tetrad” effects induced by cannabinoids which had previously been associated only with CB1R agonism [45].

## 2. Results

### 2.1. CB1R and CB2R Expression in Liver, Cortex, and Spleen

**Figure 1:**
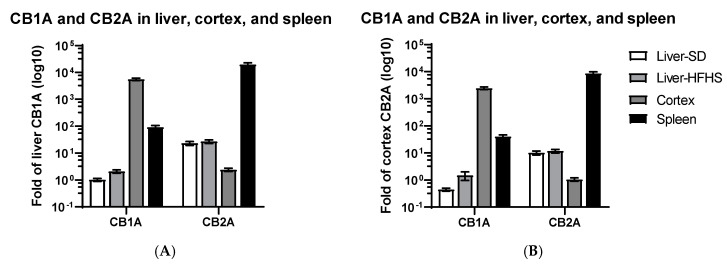
Comparison of low baseline SD (*n* = 6) and high-fat/high sugar diets (HFHS) (*n* = 6) liver cannabinoid receptors (CB1R) and cortex (*n* = 6) CB2R to high baseline cortex CB1R and spleen (*n* = 3) CB2R. SD represents standard diet and HFHS high-fat and high sugar diet. (**A**) using SD liver CB1A as a reference and (**B**) using cortex CB2A (*n* = 6) as a reference.

We first compared CB1A and CB2A isoforms that are preferentially expressed at low baseline in liver and cortex tissues, respectively [3,4]. Using low baseline standard diet (SD) liver CB1A expression as a reference, we found that high fat and high sugar diet (HFHS) significantly increased liver CB1A mRNA level 2.1 ± 0.3-fold (t_(8)_ = 2.8, *p* = 0.023), agreeing with the previous publication [46]. The high and medium baseline CB1A mRNA levels in the cortex and spleen were 5586 ± 587 and 92 ± 15-fold higher than those of the liver, respectively (Figure 1A). In contrast, using low baseline cortex CB2A as a reference, high and medium baseline CB2A mRNA levels in the spleen and liver were 8576 ± 1374 and 10.7 ± 1.2-fold higher than that of the cortex, respectively (Figure 1B). We found no significant changes of CB2R expression (t_(10)_ = 0.66, *p* = 0.527) in mouse liver with SD or HFHS diets. As expected, brain enriched CB1A expression in the cortex was 1939 ± 76-fold higher than that of cortex CB2A. We also observed that low baseline cortex CB2A mRNA level was 2.4 ± 0.3-fold higher than that of low baseline liver CB1A (Figure 1A,B). These findings indicate that the differences of high baseline cortex CB1R and spleen CB2R mRNA levels are at least three logs of dynamic ranges in comparison to the low baseline liver CB1R and cortex CB2R, respectively.

### 2.2. CB1R and CB2R Expression in Hepatocytes, Neurons, and Microglia

**Figure 2:**
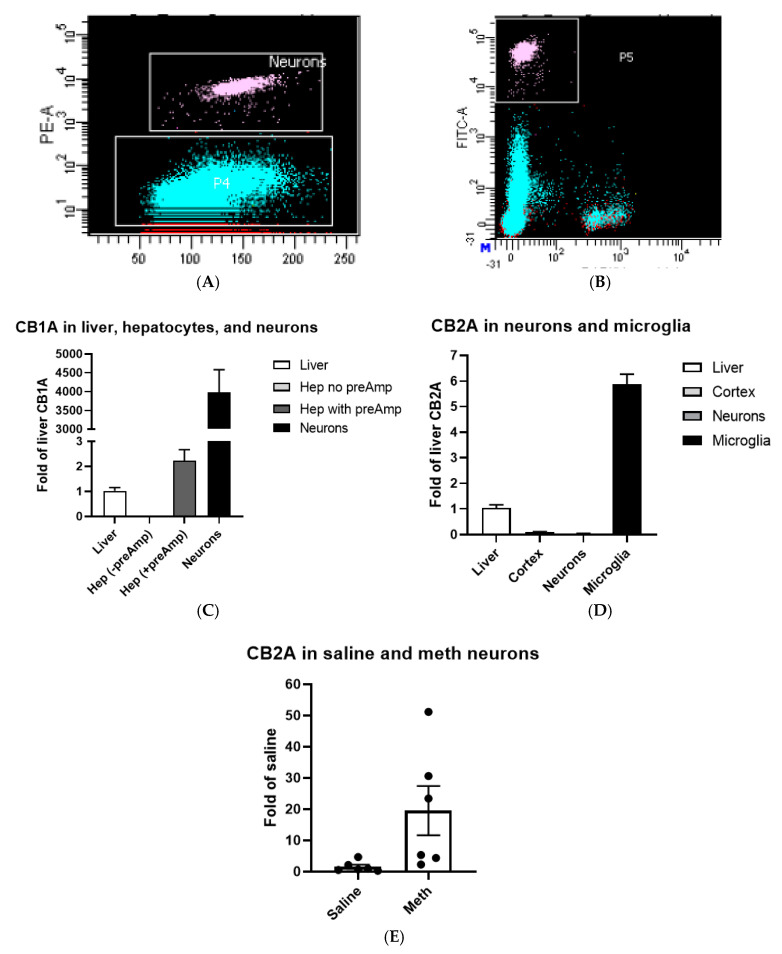
Fluorescence-activated cell sorting (FACS) of neurons and microglia. (**A**) The Y-axis is the fluorescent intensity of rat cortex neurons labeled with R-phycoerythrin (PE) conjugated NeuN antibody in logarithmic plots, and X-axis is the forward-scatter in linear scale representing cell sizes. (**B**) The Y-axis is the fluorescence intensity of transgenic Cx3c1-eGFP mouse cortex microglia in logarithmic plots, and the X-axis is the forward-scatter logarithmic plots representing cell sizes. (**C**) Comparison of low baseline CB1R expression in liver (*n* = 4), hepatocytes (*n* = 4, RT-preAmp-qPCR), and neurons (*n* = 6) using liver CB1A as a reference. (**D**) Comparison of low baseline CB2R expression in mouse cortex (*n* = 6), neurons (*n* = 6) and microglia (*n* = 4) using liver (*n* = 4) CB2A as a reference. (**E**) Comparison of CB2A expression in rat cortex neurons of saline (*n* = 6) and methamphetamine (*n* = 6) treatments.

We isolated hepatocytes from mouse liver perfused with collagenase, microglia and neurons from Cx3cr1^eGFP/+^ mouse cortex [47], and neurons of rat frontal cortex treated with saline or methamphetamine by FACS method [48]. Neurons (Figure 2A) and microglia (Figure 2B) populations from individual mouse cortex regions were well separated from other cell types and subsequently sorted and collected for RNA isolation (boxed white clusters in Figure 2A,B). We then used TaqMan RT-qPCR assay to measure microglia and neuron expression of CB1A and CB2A. CB1A mRNA level in hepatocyte was below detection of conventional TaqMan RT-qPCR, so we used TaqMan PreAmp (Thermo Fisher Scientific Inc., Waltham, MA, USA) Mix to pre-amplify hepatocyte cDNA and then measured hepatocyte CB1A mRNA level [48]. CB1A mRNA level was 4091 ± 702-fold higher in neurons than that in the liver (Figure 2C). Because hepatocyte CB1A mRNA level was too low to be lineally compared with neurons and required preamplification, CB1A mRNA level in hepatocytes was at least an order of magnitude lower than that of the liver. We found that cortex and neuron CB2A mRNA levels were 10.5 ± 2.2% and 0.5 ± 0.2% of those of liver CB2A (Figure 2D), respectively. In contrast to microglia, CB2A mRNA level was 162.4 ± 63.7-fold higher than that of neuronal CB2A (Figure 2D). We found that rat frontal cortex neuron CB2A mRNA level increased 12.7 ± 6.8-fold (t_(10)_ = 2.27, *p* = 0.047) by methamphetamine treatment in comparison with that of saline treatment (Figure 2E).

### 2.3. CB1R and CB2R Cannabinoidergic Neurons

**Figure 3:**
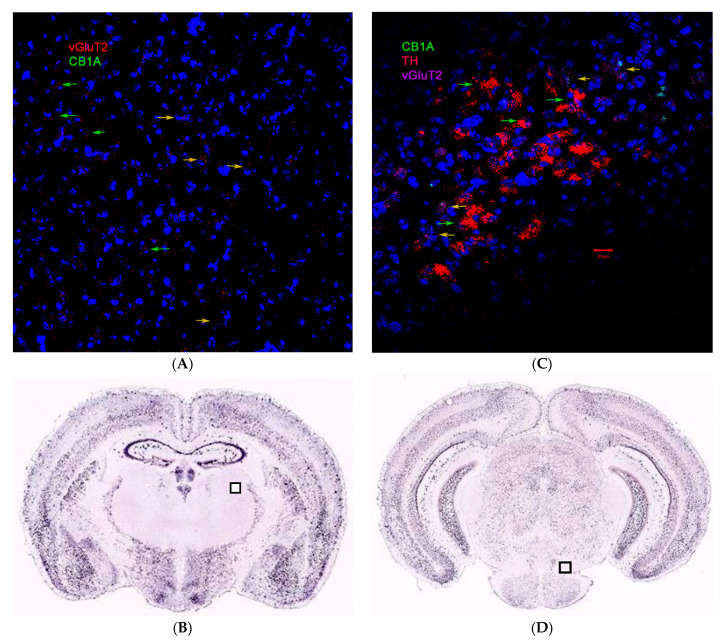
RNAscope in situ hybridization (ISH) for CB1A (**A**) brain anatomical expression in glutamatergic neurons of thalamus. Yellow arrows represent CB1A and vGluT2 co-expression in medium thalamic nucleus and green arrows represent the absence of CB1A in vGluT2 neurons. (**B**) Corresponding Allen Brain Atlas conventional CB1R ISH in mouse lateral thalamus (box). RNAscope ISH for CB1A (**C**) brain anatomical expression in dopaminergic and glutamatergic neurons of lateral VTA. Yellow arrows represent CB1A, and vGluT2 co-expression and green arrows represent the absence of CB1A in dopaminergic neurons. (**D**) Corresponding Allen Brain Atlas conventional CB1R ISH in mouse VTA (box). Scale bar is 20 μm.

Although CB1R is the most abundant GPCR in the brain, the distribution of CB1R varies drastically in different brain regions and neuron types. We could not detect CB1A RNAscope ISH signals in glutamatergic neurons of the lateral nucleus of the thalamus (green arrow in Figure 3A, Bregma −1.55 mm). We found CB1A hybridization signal in lateral VTA glutamatergic neurons; however, we could not find the CB1A signal in lateral VTA dopaminergic neurons (green arrows in Figure 3B, Bregma −3.51 mm). The Allen Brain Atlas of mouse brain coronal sections does not show traditional ISH hybridization signals of CB1R in lateral thalamus and VTA (boxed regions in Figure 3C,D, respectively) too. We previously found that CB2R ISH signals in VTA dopaminergic neurons microglia [2].

### 2.4. Generation of Cnr1^f/f^, Cnr2^f/f^, Abl-Cnr1^Δ^, and Dat-Cnr2^Δ^ Mouse Strains

**Figure 4:**
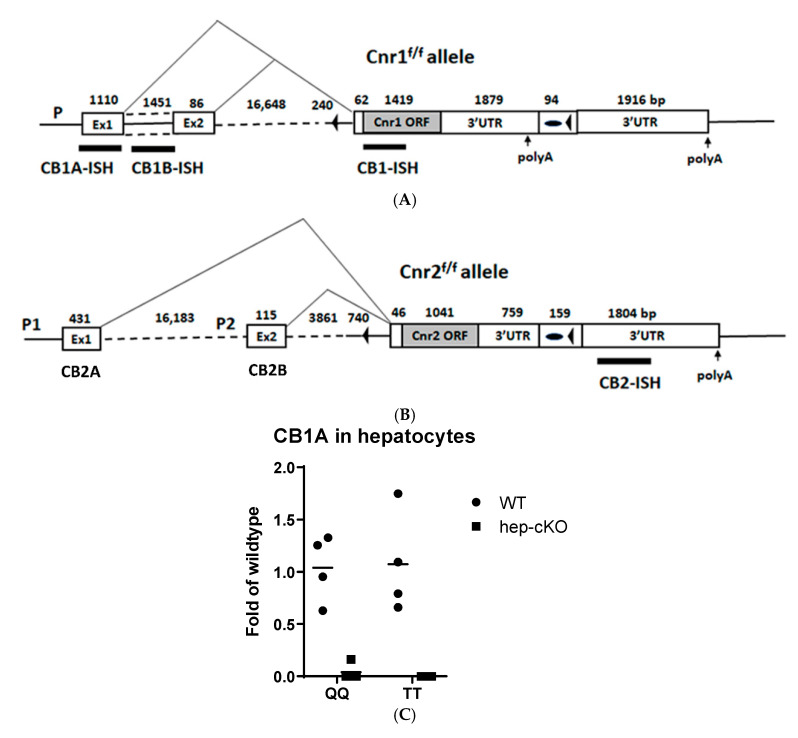

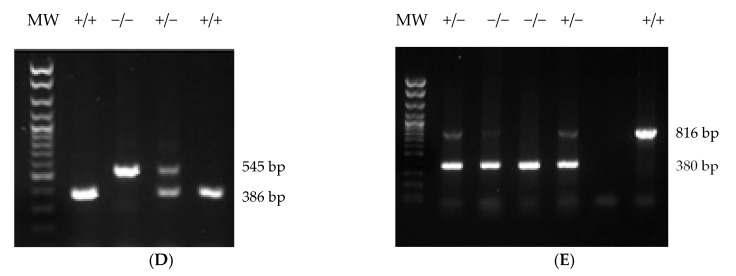
Diagrams of Cnr1^f/f^ (**A**) Cnr2^f/f^ (**B**) alleles. Open boxes represent exons and Neo-footprint, gray boxes open reading frames of Cnr1 and Cnr2, solid line introns and genomic flanking regions, triangle lines alternative splicing patterns, numbers base pairs (bp), black forward arrowheads loxP sites, and black ovals remnant FRT sites in Neo-footprint, solid bars RNAscope ISH probes, and upward arrows polyA sites. (**C**) Validation of CB1R-specific deletion in Alb-Cnr1^Δ^ hepatocytes by preAmp-RT-qPCR; Q represents QuantaBio preamp and real-time PCR mixes and T Thermo Fisher preamp and real-time PCR mixes. (**D**) Agarose gel of Cnr2 floxed mice: mutant is 545 bp, wild type 386 bp, and the heterozygous 545 bp and 386 bp. (**E**) Agarose gel of Cx3cr1-Cre mice: mutant is 380 bp, wild type 816 bp, and the heterozygous 380 bp and 816 bp. MW represents the molecular weight marker.

We designed Cnr1-loxP-FRT-Neo, and Cnr2-loxP-FRT-Neo constructs in which the coding exon of *Cnr1* and *Cnr2,* including the exon splicing acceptor sites, was flanked with loxP sites and the Neo selection marker gene was flanked by FRT sites. Upon FLP recombination in the FLP-deleter ES line, the Neo gene was eliminated, and a distal loxP and a proximal loxP/FRT footprint remain in the genomes of Cnr1^f/f^ and Cnr2^f/f^ mouse strains. Upon Cre recombination in Cre-deleter transgenic mice, the *Cnr1* and *Cnr2* splicing acceptor sites and entire ORFs were deleted in Abl-Cnr1^Δ^, and Dat-Cnr2^Δ^ mouse strains, respectively. Figure 4A shows the diagram of the Cnr1^f/f^ allele; the distal loxP site is localized upstream (240 bp) of the splicing acceptor site that is 62 bp upstream of the *Cnr1* translational initiation codon. The proximal loxP site is localized in the downstream (1879 bp) of the stop codon and the first polyA signal site, but upstream of the second polyA site in *Cnr1* 3′ UTR so that the splicing site, the entire ORF and the first polyA signal site, and most of Neo-footprint (94 bp) are deleted upon Cre recombination, and the partial 3′UTR sequence (1916 bp) remains in Abl-Cnr1^Δ^ mouse strain. Figure 4B shows the diagram of the Cnr2^f/f^ allele; the distal loxP site is localized upstream (740 bp) of the splicing acceptor site that is 46 bp upstream of the *Cnr2* translation initiation codon. The proximal loxP site is localized in the downstream (759 bp) of the stop codon in 3′ UTR so that the splicing site and the entire open reading frame, including the partial 3′ UTR and most of Neo-footprint (159 bp), are deleted upon Dat-*Cre* recombination, and the partial 3′ UTR sequence (1804 bp) remains in Dat-Cnr2^Δ^ mouse strains. Sanger sequencing confirmed the sequences of Cnr1^f/f^ and Cnr2^f/f^ alleles, loxP sites, and Neo-footprints. RT-preAmp-qPCR TaqMan assay demonstrated that CB1R was specifically deleted in hepatocytes of Abl-Cnr1^Δ^ strain (Figure 4C), and RNAscope ISH experiment demonstrated that CB2R was specifically deleted in Dat-Cnr2^Δ^ dopaminergic neurons [2] and Cx3cr1-Cnr2 ^Δ^ microglia (Figure 4D,E).

### 2.5. Tetrad and Alcohol Effects Following Deletion of CB2Rs in Dopamine Neurons and Microglia

**Figure 5:**
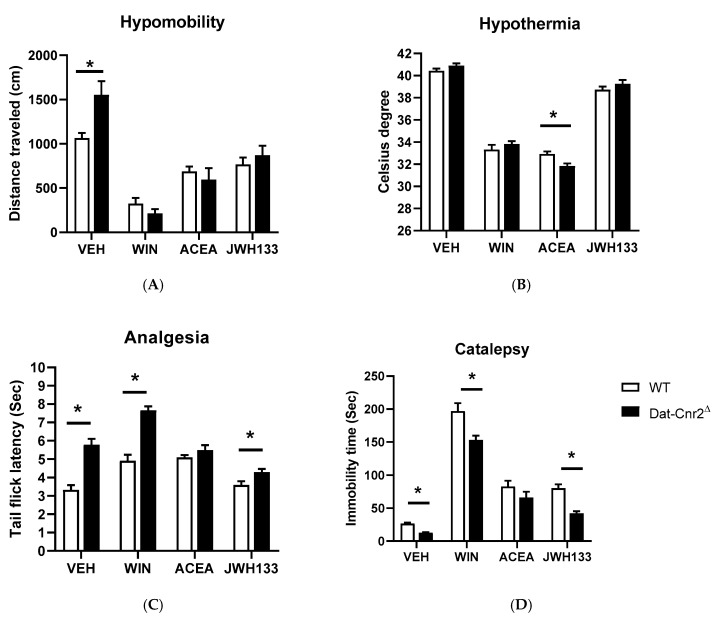
CB2R-mediated behaviors in the tetrad effects in DAT-Cnr2^Δ^ mice. The effects of CB1R, CB2R and mixed CB1R and CB2R ligand, WIN 55212-2 (3.0 mg/kg), ACEA (1.0 mg/kg) and JWH133 (20 mg/kg) in the tetrad effects. (**A**) Hypomobility; (**B**) hypothermia; (**C**) analgesia (tail-flick nociception test and a similar result in the hot plate test); (**D**) catalepsy. Asterisks represent *p* < 0.05.

**Figure 6:**
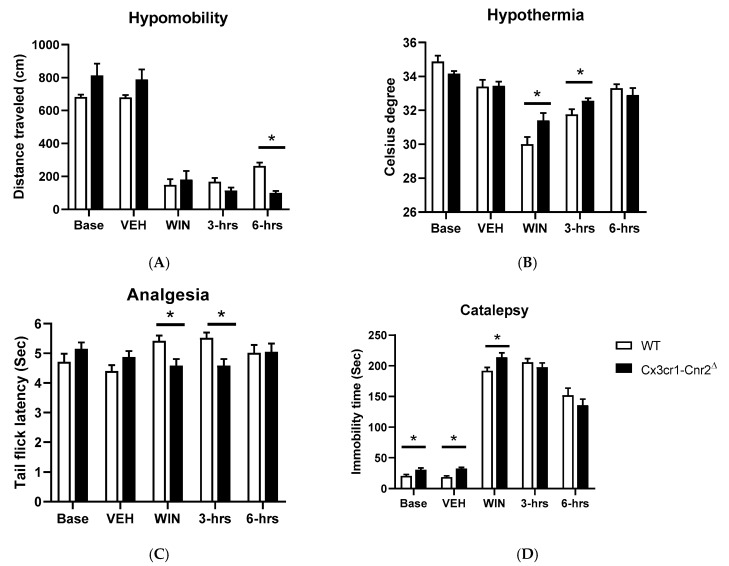
CB2R-mediated behaviors in the tetrad effects in WT, Cx3cr1-Cnr2 mice. The effects of the selected dose of the mixed CB1R and CB2R ligand WIN 55212-2 (3.0 mg/kg) in the tetrad effects. (**A**) Hypomobility; (**B**) hypothermia; (**C**) analgesia (tail-flick nociception test and a similar result in the hot plate test); (**D**) catalepsy. Asterisks represent *p* < 0.05.

**Figure 7:**
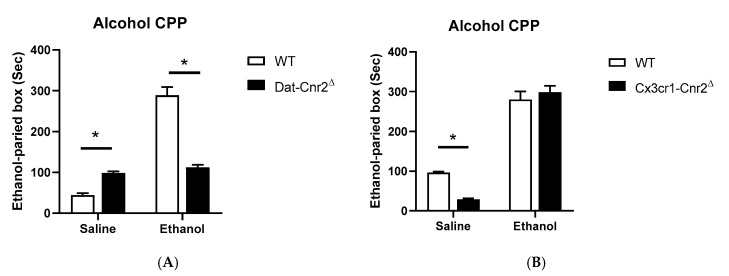
CB2R modulation of alcohol preference in Alcohol 8%-induced conditioned place preference in WT and Cx3cr1-Cnr2^Δ^ mice, but not in DAT-Cnr2^Δ^ mice. (**A**) WT and DAT-Cnr2^Δ^ mice; (**B**) WT and Cx3cr1-Cnr2^Δ^ mice. Asterisks represent *p* < 0.05.

It was previously considered that the mouse tetrad effects induced by cannabinoid ligands such as Δ^9^-THC were mediated by CB1R activation [1]. Contrary to this long-standing notion that the characteristic profile of catalepsy, hypothermia, antinociception, and hypomobility was mainly by CB1R agonism, our data using the Cre-loxP technology to generate mice with cell-specific deletion of CB2Rs in dopamine neurons and microglia reveals that CB2Rs are involved in the tetrad effects. We compared changes of the main effects of drugs and genotypes in DAT-Cnr2^Δ^ mice previously reported in our labs [2,49] and the main effects of drugs and genotypes in Cx3cr1-Cnr2^Δ^ mice by two-way ANOVA (Table 1). Parallel results showed hyper-locomotor activity phenotype of the DAT-Cnr2^Δ^ mice in vehicle-treated but disappeared in CB1R and CB2R agonists-treated mice (Figure 5A). Hypothermia was significantly different between WT and DAT-Cnr2^Δ^ mice following ACEA treatment and not significant in WIN 55212-2 or JWH133 mice (Figure 5B). DAT-Cnr2^Δ^ mice showed enhanced analgesia (Figure 5C) and reduced catalepsy (Figure 5D) in vehicle, mixed or selective CB2R agonists of WIN 55212-2 and JWH133, respectively, except after CB1R selective agonist ACEA treatment. In contrast, Cx3cr1-Cnr2^Δ^ mice did not show hyper-locomotor activity in vehicle treatment. However, WIN 55212-2-induced hypomobility was significant after a 6 h treatment in comparison to WT mice (Figure 6A). The hypothermia was significantly different between Cx3cr1-Cnr2^Δ^ and WT mice in WIN 55212-2 treatment at zero and after 3-h (Figure 6B). Cx3cr1-Cnr2^Δ^ mice showed reduced analgesia (Figure 6C) in WIN 55212-2 treatment at zero and after 3-h (Figure 6D). We observed that Cx3cr1-Cnr2^Δ^ mice showed enhanced catalepsy in baseline, vehicle, and WIN 55212-2 treatments, and the enhanced catalepsy phenotype disappeared after the treatment in 3- and 6-h (Figure 6D). We found that the alcohol preference ratio was significantly changed in the main effects of alcohol and genotypes in DAT-Cnr2^Δ^ mice [2] and in the main effects of genotype in Cx3cr1-Cnr2^Δ^ mice by two-way ANOVA (Table 1). DAT-Cnr2^Δ^ mice did not condition to alcohol [2], and the alcohol CPP was significantly lower in DAT-Cnr2^Δ^ compared to WT mice (Figure 7A); however, Cx3cr1-Cnr2^Δ^ mice conditioned to alcohol preference did not differ from WT mice (Figure 7B). These results demonstrate the functional neuronal or microglia expression of CB2Rs that are involved differentially in the tetrad test and alcohol preference.

## 3. Discussion

We demonstrate that low basal and inducible CB1R and CB2R have significant functional implications in their dually and singly expressed cell types, and CB2R in neurons and microglia are differentially involved in the “tetrad” and alcohol preference effects. We showed that CB2R expression in neurons was higher than that of hepatocyte CB1R, for which function has been demonstrated [37,50].

The differential physiological roles of cannabinoid receptors depend on their cell-type-specific localization. Cre-lox recombination—a recombinase technology has been used for deletion of CB1Rs, and many conditional *Cnr1* mutant mice have been produced [24,29], with significant knowledge and improved functional roles of CB1Rs in mouse models of human disorders. However, CB2R cell-type-specific functional studies lagged those of CB1Rs. Hence many features of CB2Rs function, variation and impact on behavior remain poorly defined compared to CB1Rs. Using the Cre-*loxP* system to generate cell-type-specific deletion of CBRs in the hepatocyte, microglia, and dopamine neurons, we show that the application of Cre-loxP recombinant mouse strains unravels hitherto unknown physiological, behavioral and signaling mechanisms associated with CB2R function. For example, the upregulation of CB1R by HFHS diet in hepatocytes has an inverse relationship with the downregulation of CB2R by lipid overload in the co-expressed hepatocytes [51,52]. Therefore, we isolated adult rodent hepatocytes, neurons, and microglia to compare CB2R expression to those of CB1R. We provide direct evidence of low baseline CB2R expression in neurons that is higher than that of low baseline hepatocyte CB1R for which their expression and function have not been controversial [37,50]. We used TaqMan probes targeting CB1A and CB2A-specific isoforms that are expressed at very low levels in hepatocytes and neurons [3], respectively. TaqMan RT-qPCR and RT-preAmp-qPCR assays are better suited for low baseline detection because it has 9 and >12 logs of dynamic range measurements, respectively, in contrast to 2 logs of dynamic ranges of traditional end-point PCR fragment analysis [10]. We observed that to detect neuron CB2R by RT-qPCR without preamplification, the <Ct 18 values of endogenous control β-actin was needed. We found that microglia CB2R mRNA is much higher than that of neuron CB2R, partially agreeing with Cnr2-GFP reporter knock-in mouse strains [12,13] in which the brain’s GFP signals were observed in microglia but not in neurons. We argue that the 33 kD GFP signals in transgenic mice have a low dynamic range of output [53], and the GFP replacement of *Cnr2* ORF or knock-in the 3′ UTR of *Cnr2* in the transgenic mice might compromise translational control of low baseline neuron CB2R mRNA [54]. However, the Cnr2-GFP signal could be detected in mouse retina neurons of the inner nuclear layer and ganglia cell layer treated with endotoxin lipopolysaccharides (LPS) [55], indicating the inducible nature of neuronal CB2R mRNA for which the translational product could have a long half-life [56]. To our knowledge, there is no published work on CB1R GFP-reporter or radio-ligand-binding signals in low baseline CB1R expressed hepatocytes. We previously found that microglia CB2R mRNA level is 357 ± 77-fold higher than that of neurons in primary cell cultures of rat fetus cortex [3]. We now demonstrate that microglia CB2R mRNA level was 162 ± 63-fold higher than that of neurons in adult mouse brain. Since CB2R in microglia is also inducible [57,58], the purified microglia CB2R expression may not reflect that of naïve microglia. Our finding of increased expression of neuron CB2R by methamphetamine treatment agrees with previous findings of neuron CB2R upregulation by brain stressors such as cocaine [59], traumatic brain injury [60], seizure [61], and ischemia [62].

Neurons are the most heterogeneous cell types that can be divided into different neuronal types depending on their responses to neurotransmitters [63], and a neuron type can be further divided into different subpopulations depending on neurotransmitter receptor multiplexing [64]. Our study shows that cannabinoidergic neurons can be divided into different neuron types and subpopulations depending on CB1R and CB2R singly and dually cell-type-specific co-expression networks interacting with other neurotransmitter receptors and transporters [64,65]. To avoid the CB2R antibody nonspecificity issue, we used ultrasensitive RNAscope ISH [4], behavioral pharmacology [66], and electrophysiology [67] to study functional neuronal CB2R. Traditional ISH is based on a 1:1 ratio of the probe and target hybridization [68], and RNAscope ISH is based on branched DNA amplification 8000:1 ratio of the probe and target hybridization. The difference is like light and electron visions of microscopes. The advantage of RNAscope ISH is obvious not only in the signal amplification but also in noise reduction due to simultaneous full hybridization of 10–20 ZZ oligo pairs to 0.5–1.0 kb-specific region of mRNA [69] that is well suited for low baseline neuron CB2R detection [59]. CB1R and CB2R can also form a heterodimer in their co-expressed neurons in the pallidothalamic nucleus, and reduction of their expression is observed in Parkinson’s disease [70]. We found the presence of cannabinoidergic non-overlapping CB1R and CB2R neuron populations in glutamatergic neurons in the lateral nucleus of the thalamus and dopaminergic neurons in VTA, and they might multiplex with other neurotransmitter receptors on demand for CB1R-mediated cell-type-specific depolarization-induced suppression of inhibition (DSI) [71] and excitation (DSE) [72] and CB2R-mediated hyperpolarization [38,39]. Transiently-induced CB2A using neuron preferential alternative promoter might pivot the receptor to axon guidance and synapse growth [73,74], and critical intracellular organelles such as mitochondria, endoplasmic reticulum, the autophagosome, and lysosomes [75] and subside when stressors are removed. A similar case is a sodium-iodide symporter (NIS), which is a high baseline expressed gene in follicular cells of the thyroid gland [76] and a low baseline in nubile mammary glands, but drastically upregulated on demand in alveolar cells of healthy lactating mammary gland of mother to meet baby needs for biosynthesis of thyroid hormone and subside to low baseline after lactation [77]. Low baseline neuron CB2R regulation is dynamic and time-dependent [62], and we took snapshots of hepatocyte CB1R and neuron CB2R by RT-qPCR, RT-preAmp-qPCR, and RNAscope methods in vitro. To understand CB1R and CB2R pharmacological, biochemical, and behavioral functions in vivo, we propose to use Cre-loxP recombination to delete CB1R, CB2R, or both in specific brain regions and cell types of interests.

To investigate peripheral CB1R function, we generated Cnr1^f/f^ mouse strain [78] that was crossed with Abl-Cre-deleter strain to produce hepatocyte-specific cKO of Abl-Cnr1^Δ^ mice, and we found that deletion of CB1R in hepatocyte-protected concanavalin A-induced liver damage (Kim et al. in revision), crossed with MIP-Cre-deleter strain to produce MIP-Cnr1^Δ^ mice and we found that deletion of CB1R in adult pancreatic β-cells reduced diabetes-induced inflammation [78], crossed with Acta-Cre-deleter strain to produce Acta-Cnr1^Δ^ mice and we found that deletion of CB1R in myocyte prevented diet-induced and age-induced insulin resistance [79].

To investigate neuron and microglia CB2R function, we generated Cnr2^f/f^ mouse strain that was crossed with Dat-Cre-deleter strain [80] and Cx3cr1-Cre-deleter strain to produce dopaminergic neuron-specific cKO of Dat-Cnr2^Δ^ mice [2]. In the follow-up, we crossed the Cnr2^f/f^ mouse strain with microglia Cx3cr1-Cre-deleter strain to produce microglia-specific cKO of Cx3cr1-Cnr2^Δ^. We found that the deletion of CB2R in dopamine neurons enhances motor activities, modulates anxiety, and dopamine-related effects of alcohol and psychostimulants [2,81]. In contrast, the deletion of CB2Rs did not differ from the WT in their motor activities. However, both DAT-Cnr2 and the Cx3cr1-Cnr2 cKO mice revealed that CB2Rs are involved in the “tetrad” effects, but the alcohol preference ratio was significantly higher in the Cx3cr1-Cnr2 and WT than that of DAT-Cnr2 cKO mice that consumed less alcohol. In support of our finding of neuron CB2R function, European labs generated principle neuron-specific Syn-Cnr2^Δ^ mouse strain by crossing Cnr2^f/f^ strain with Syn-Cre-deleter mouse strain [38]. Electrophysiological and behavioral studies of Syn-Cnr2^Δ^ mice found that the activation of neuronal CB2R enhanced a long-lasting membrane potential hyperpolarization by activating autonomous slow self-inhibition in neurons of hippocampus and cortex [38,39] and reduced neuropathic pain and anxiety [40]. By crossing the Cnr2^f/f^ strain with monocyte-specific LysM-Cre-deleter mice to generate LysM-Cnr2^Δ^ cKO mice [40], the authors found that both lymphocyte and neuron CB2Rs participate in coordinated functions in neuropathic pain. An intriguing observation is that lymphocytes participate in CB2R induced antinociception by infiltration into dorsal root ganglia (DRG) of the injured nerve and transferred their high gradient CB2R to low gradient neuron CB2R [40]. It is plausible that CB2R could be transferred from high gradient microglia to low gradient neurons on demand for brain damage control.

To understand complex peripheral and central cell-type functions of cannabinoid receptors, double Cnr1^f/f^ and Cnr2^f/f^ mouse strain could be produced genetically and crossed with cell-type-specific Cre-deleter mouse strains to study functions of CB1R and CB2R co-expressed cell types [70,82,83]. On the other hand, genetically engineered AAV-GFP-Cre viruses could be delivered to specific brain regions of Cnr1^f/f^, Cnr2^f/f^, and Cnr1^f/f^/Cnr2^f/f^ strains by stereotaxic microinjection to delete CB1R, CB2R, or both [84,85]. Furthermore, inducible knockout of Cnr1^f/f^, Cnr2^f/f^, and Cnr1^f/f^/Cnr2^f/f^ loci can be achieved by microinjection of cell-type-specific promoter linked AAV-GFP-Cre virus to study CB1R and CB2R interactions with other neuron types that underline neurocircuitry and behavior [86,87]. We designed TaqMan duplex assays to genotype wildtype, heterozygous, and homozygous Cnr1^f/f^, Cnr2^f/f^, Abl-Cnr1^Δ^, and Dat-Cnr2^Δ^ mice and the duplex assay may be extended to triplex and quadruplex assay to save time and reagents. Cell-type-specific cKO mice of CB1R, CB2R, or both will promote understanding of CBRs not only in the high baseline but also low baseline CB1R and CB2R expressing cell types in vivo and in real time. In the current COVID-19 pandemic period, the application of CB2R agonists for therapeutic treatment of COVID-19 could be explored for their anti-inflammation effects [88] and potential interaction to SARS-CoV-2 spike glycoprotein receptor-binding domains (RBDs) where linoleic acid binds and exerts allosteric inhibition [89].

We provide evidence that cell-type-specific expression of cannabinoid CB2 receptor subtypes in brain dopamine neurons and microglia reveals that low basal and inducible expression of brain CB2R is involved in alcohol consumption and in the “tetrad” effects induced by cannabinoids, which had previously been associated only with CB1R agonism. Therefore, *Cnr1* and *Cnr2* cKO mice with cell-type-specific deletions [90] in cannabinoid receptor-expressing and “non-expressing” cells [8], as well as intracellular organelles [91,92] is deepening our understanding of the ECS system. This will contribute to the development of cannabinoid medicines for chronic diseases such as Alzheimer’s disease, neuropathic pain, diabetes, obesity, nephrotic syndrome, autoimmune diseases, post-traumatic stress disorder, osteoporosis, fibrosis, anxiety disorder, and addiction.

## 4. Materials and Methods

### 4.1. Animals and Isolation of Hepatocytes, Neurons, and Microglia

All animal care and experimental procedures performed according to US National Institutes of Health guidelines and were approved by Animal Care and Use Committees of NIA (478-LCI-2021, approved on 17 April 2018), and Department of Biology, William Paterson University (D16-00879 (A4681-1) on 24 October 2018). Male C57BL/6 J mice were fed control and a high-fat/high sugar diets (HFHS) for 16 weeks [93], and then perfused with collagenase through portal vein and hepatocytes were isolated from the perfused and digested liver by pipette dispersion and filtration with cell strainer [94]. Male Sprague-Dawley rats were injected with saline (1 mL/kg, i.p.) or (+)-methamphetamine-HCl (NIDA drug supply; 5 mg/kg, i.p.), and 3 h after the injection, the cortex were dissected, and neurons were isolated by fluorescence-activated cell sorting (FACS) labeled by neuron-specific selection marker NEUN antibody conjugated with phycoerythrin (PE) fluorophore. Male CX3CR1^eGFP/+^ mice were used for cortex dissection, and eGFP-positive microglia and NEUN-labeled positive neurons were isolated by FACS as described by our previous work [47,48,95].

### 4.2. RNA Isolation and TaqMan RT-qPCR, RT-preAmp-qPCR Assays

Total RNAs were isolated from brain regions and peripheral tissues using the TRIzol Reagent. Single strand cDNAs were synthesized using qScript XLT cDNA SuperMix (Quantabio, Beverly, MA, USA, #95161-500). Rodent isoform CB1A [3] and CB2A [4] FAM-labeled probes and endogenous control VIC-labeled *Actb* probe (Thermo Fisher Scientific Inc., Waltham, MA, USA, #4352341E) were used for TaqMan RT-qPCR. To validate hepatocyte CB1R detection, TaqMan PreAmp Master Mix Kit (Thermo Fisher Scientific Inc., Waltham, MA, USA, #4391128) or PerfeCTa PreAmp SuperMix (Quantabio, Beverly, MA, USA, #95146-005) was used for cDNA preamplification using 80 nM of forward and reverse primer sets [48]. cDNAs were pre-amplified using the program: 95 °C hold for 10 min and then 10 cycles of denaturation at 90 °C for 15 s and annealing and extension at 60 °C for 4 min. Duplex PCR assays containing both the target and endogenous control TaqMan probes were carried out with Advanced TaqMan Fast PCR Master Mix (Thermo Fisher Scientific Inc., Waltham, MA, USA, #4444556) or PerfeCTa Multiplex qPCR ToughMix (Quantabio, Beverly, MA, USA, #95147-250) in StepOnePlus instrument using a default thermo-cycling program. The relative fold change is calculated using the formula: 2^(−△△Ct)^ [48].

### 4.3. RNAscope In Situ Hybridization (ISH)

For RNAscope in situ hybridization (ISH) experiment, mCB1R (20 ZZ pairs targeting 530–1458 of NM_007726 within coding exons), vGluT2 RNAscope probe (Mm-Slc17a6-C3: Cat# 319,171-C3, targeting 1986–2998 bp of NM_080853.3), TH-specific RNAscope probe (Mm-Th-C2: Cat# 317,621-C2, targeting 483–1603 bp of NM_009377.1) and custom probes mCB1A (16 ZZ pairs targeting 104–1097 of AK163855 within exon 1) were ordered from Advanced Cell Diagnostics (ACDbio, Newark, CA, USA) and their hybridization positions were shown graphically (Figure 6A,B). We carried out duplex and triplex ISH of mouse frozen brain sections following the manufacturer’s protocol with slight modifications [3]. We downloaded conventional ISH mouse brain coronal section images [96] of *Cnr1* from Allen Brain Atlas (https://mouse.brain-map.org/) as a comparison.

### 4.4. Generation Cnr1^f/f^ and Cnr2^f/f^ Mouse Strains

We designed and produced Cnr1-floxed-Neo-FRT and Cnr2-floxed-Neo-FRT constructs in which the complete *Cnr1* or *Cnr2* ORFs and the coding exon splicing acceptor sites are flanked by loxP sites in collaboration with Ingenious Targeting Laboratory (https://www.genetargeting.com/). After transfection of the constructs into murine (strain 129) embryonic cells (ESCs), the Neo marker was deleted in FLP-deleter ESC culture, and the selected ES clones were injected into mouse blastocoel of blastocysts to produce Cnr1^f/f,^ and Cnr2^f/f^ mouse strains and the alleles were sequenced by Sanger method.

### 4.5. Generation of Abl-Cnr1^Δ^, Dat-Cnr2^Δ^, and Cx3cr1-Cnr2^Δ^ Recombinant Strains

Hepatocyte-specific Cre-deleter and microglia-specific Cre-deleter mice were ordered from The Jackson Laboratory (https://www.jax.org/): Alb-Cre mice B6.Cg-Speer6-ps1Tg(Alb-cre)21Mgn/J (stock# 003,574) and Cx3cr1-Cre mice (B6J.B6N(Cg)-Cx3cr1tm1.1(cre)Jung/J). Dopaminergic neuron-specific Cre-deleter mice were generated at NIDA with IRES-Cre cassette knocking into 3′ UTR of *Slc6A3* gene in order not to disrupt dopamine transporter function in the transgenic mouse [80]. Cnr1^f/f^ and Cnr2^f/f^ mice were crossed with these Cre-deleter mice at animal facilities of Intramural Research Programs (IRPs) of the National Institute on Aging (NIA) and Department of Biology, William Paterson University, respectively, to generate Abl-Cnr1^Δ^, Cx3cr1-Cnr2^Δ^, and Dat-Cnr2^Δ^ mice that were bred for more than10 generations on the background of C57BL/6 J mice. Mouse tail genomic DNA was isolated using QIAamp DNA Mini Kit according to the manufacturer’s protocol. TaqMan probes were designed using Primer Express v3.0.1 software (Thermo Fisher Inc., Waltham, MA, USA) for genome typing wildtype, heterozygous, and homozygous Cnr1^f/f^ and Cnr2^f/f^ mice, as well as Abl-Cnr1^Δ^ and Dat-Cnr2^Δ^ mutant mouse strains. Real-time duplex genotyping assays were carried out with Advanced TaqMan Fast PCR Master Mix (Thermo Fisher Inc., Waltham, MA, USA, #4444556) in which specific wildtype FAM- and mutant VIC-labeled 3′-minor groove binder-DNA TaqMan probes (Table 2) were mixed with 1 μg of genomic DNA template in a single well to determine wildtype, heterozygous, and mutant alleles simultaneously in StepOnePlus instrument using a default thermo-cycling program. The cycle threshold (Ct) of positive signals were usually <30 Ct values. Cx3cr1-Cnr2^Δ^ mutant mouse genotypes were analyzed by PCR fragment analysis in 1% agarose gel stained with SYBR green (Table 2).

### 4.6. Behavioral Assays

In a battery of behavioral test systems, adult mice with the deletion of CB2Rs in microglia and dopamine neurons were evaluated in models of CNS function in comparison to WT controls with a C57BL/6 background. The performances in motor function tests were conducted using the activity monitors. Cannabinoid-induced “tetrad” effects were analyzed using the tetrad effects following our published protocols [2]. The vehicle used was tween-80: DMSO: saline in a ratio of 1:2:7, mixed agonist WIN 55212-2, CB1R and CB2R selective agonist Arachidonyl-2′-chloroethylamide (ACEA) and JWH133, respectively, were used for i.p. injection for the tetrad effects. Modification of 8% alcohol consumption and preference in the Dat-Cnr2^Δ^ and Cx3cr1-Cnr2^Δ^ male mice and their WT male controls were assessed. The open-field test was used for the assessment of general activity. The classical cannabinoid-induced catalepsy, hypothermia, antinociception, and suppression of spontaneous locomotor activity are referred to as tetrad effects. Groups of CB2R cKO and WT mice were evaluated in the tetrad effects with and without administration of vehicle, and cannabinoid ligands were determined. All tests of alcohol consumption were investigated using the two-fluid bottle choice. The ratio of alcohol to water consumed, and the total fluid consumption was calculated to obtain a conditioned placement preference (CPP) ratio. These routine behavioral assays have been described elsewhere [2,40,49]. The animal experiments were performed in the laboratory of Professor Emmanuel Onaivi at William Paterson University in NJ. The experimental procedures followed the Guide for the care and use of laboratory animals and were approved by the William Paterson University animal care and use committee. Statistical analysis was performed with Prism version 8.0 (GraphPad Software Inc., La Jolla, CA, USA). The number of mice in each group studied in Tetrad effects, and alcohol CPP tests were 8 and 10, respectively. Behavioral data are presented as mean ± SEM and calculated by repeated measures and mixed-effects model of two-way analysis of variance (ANOVA) followed by Sidak post hoc test for multiple comparisons. *P* values of <0.05 were defined as significant.

## Figures and Tables

**Table 1:** Results of the statistical analysis of the tetrad effects and alcohol preference ratio of wild type (WT), DAT-Cnr2^Δ^ and Cx3cr1-Cnr^Δ^ mice by two-way ANOVA. ^a^ Two-way ANOVA, matched samples, ^b^ Two-way ANOVA, mixed-effects model (tail-flick WT WIN-6-h 2 mouse data are missing and immobility WT WIN-3-h 1 mouse data are missing).

Dat-Cnr2^Δ^	Main Effect of Drugs	Main Effect of Genotypes	Interaction
^a^ Locomotion	F (1, 14) = 2.583 *p* = 0.1303	F (2.217, 31.04) = 39.01 *p* < 0.0001	F (3, 42) = 4.017 *p* = 0.0134
^a^ Temperature	F (1, 14) = 0.2189 *p* = 0.6471	F (2.481, 34.73) = 435.1 *p* < 0.0001	F (3, 42) = 4.240 *p* = 0.0105
^a^ Nociception	F (1, 14) = 62.10 *p* < 0.0001	F (1.820, 25.48) = 34.80 *p* < 0.0001	F (3, 42) = 12.34 *p* < 0.0001
^a^ Catalepsy	F (1, 14) = 31.49 *p* < 0.0001	F (2.151, 30.12) = 181.8 *p* < 0.0001	F (3, 42) = 2.382 *p* = 0.0830
^a^ Ethanol 8%	F (1, 18) = 27.88 *p* < 0.0001	F (1, 18) = 143.7 *p* < 0.0001	F (1, 18) = 114.7 *p* < 0.0001
**Cx3cr1-Cnr2^Δ^**			
^a^ Locomotion	F (1, 14) = 0.09896 *p* = 0.7577	F (2.058, 28.81) = 191.3 *p* < 0.0001	F (4, 56) = 7.032 *p* = 0.0001
^a^ Temperature	F (1, 14) = 2.516 *p* = 0.1350	F (2.567, 35.94) = 33.74 *p* < 0.0001	F (4, 56) = 3.120 *p* = 0.0218
^b^ Nociception	F (1, 69) = 1.196 *p* > 0.05	F (2.221, 38.31) = 380.4 *p* < 0.0001	F (4, 69) = 2.999 *p* < 0.05
^b^ Catalepsy	F (1, 14) = 0.6054 *p* > 0.05	F (2.387, 32.22) = 1.676 *p* > 0.05	F (4, 54) = 6.708 *p* < 0.05
^a^ Ethanol 8%	F (1, 18) = 3.369 *p* = 0.0830	F (1, 18) = 317.4 *p* < 0.0001	F (1, 18) = 11.35 *p* = 0.0034

**Table 2:** Nucleotide sequences of TaqMan probes and primers for PCR fragments for genotyping of the transgenic mice.

Alleles	TaqMan Probe or PCR Fragment	Forward Primer	Reverse Primer
Cnr1-wt	CATCTGTTGGTGATTTCT(FAM)	CCTAAGAACTGCATGGCATGAAG	GCTGGGAACCCCAAATGGT
Cnr1-flox	CTAGCATCTGTTGGAGTGTAC(VIC)	CCTAAGAACTGCATGGCATGAAG	GGAACTTCGCTAGACTAGTACGC
Cnr2-wt	AGTCTTCAGAGAACTCT(FAM)	GCTGGGTTCACTGGAGGTACA	ACACAGCAAAATGTCACAAGGAA
Cnr2-flox	AGTCTTCAATTGCGTACGTT(VIC)	GCTGGGTTCACTGGAGGTACA	CGCGACACGGACACAATC
Cnr2-wt	386 bp PCR FRAGMENT	GGTCAAGAATTATGATGCCCTAAGGACC	CCCAACTCCTTCTGCTTATCCTTCAGG
Cnr2-flox	545 bp PCR FRAGMENT	GGTCAAGAATTATGATGCCCTAAGGACC	CCCAACTCCTTCTGCTTATCCTTCAGG
Abl-wt	CCTGTCATGCCCACACAAATCTCTCC(FAM)	GCTGTCATCTCTTGTGGGCTGT	ACTCATGGGAGCTGCTGGTTC
Abl-Cre	CTATCAACCCCGGGATCC(VIC)	AGCGAGTCTTTCTGCACACA	GCTGCAGGTCGACTCTAGATC
Dat-wt	AGATCACAAAGGAAACC(FAM)	GCCAGCTGGGCCATCTC	AAGTGGCCCTCCTTTCTTGAC
Dat-Cre	CCCCCCTAACGTTACT(VIC)	GTTGGTGTAAAGTGGAAGGAGACA	CGCACACCGGCCTTATTC
Cx3cr1-wt	380 bp PCR FRAGMENT	AGCTCACGACTGCCTTCTTC	GCAGGGAAATCTGATGCAAG
Cx3cr1-Cre	816 bp PCR FRAGMENT	GACATTTGCCTTGCTGGAC	GCAGGGAAATCTGATGCAAG
Cre	GGTTAGCACCGCAGG(VIC)	TTAATCCATATTGGCAGAACGAAAACG	CAGGCTAAGTGCCTTCTCTACA

## Abbreviations

ACEA	Aarachidonyl 2′ chloroethylamide
ANOVA	Analysis of variance
CB1R	Cannabinoid type 1 receptor
CB2R	Cannabinoid type 2 receptor
*Cnr1*	Mouse cannabinoid receptor 1 gene symbol
*Cnr2*	Mouse cannabinoid receptor 2 gene symbol
cKO	Conditional knockout
Cnr1^ff^	Cannabinoid receptor 1 floxed
Cnr2^ff^	Cannabinoid receptor 2z floxed
CPP	Conditioned place preference
Ct	Cycle threshold
Cx3cr1	Chemokine receptor1
DAT	Dopamine transporter
DSI	Depolarization-induced suppression of inhibition
DSE	Depolarization-induced suppression of excitation
DMSO	Dimethyl sulfoxide
ECS	Endocannabinoid system
eGFP	Enhanced green fluorescence protein
ESCs	Embryonic stem cells
FACS	Fluorescence-activated cell sorting
FITC	Fluorescein isothiocyanate
gKO	Germline knockout
GFP	Green fluorescence protein
GPCR	G-protein coupled receptor
Hep-cKO	Hepatocyte conditional knockout
HFHS	High-fat high sugar
ISH	In situ hybridization
NIS	Sodium iodide transporter
ORF	Open reading frame
PE	Phycoerythrin
RT–PCR	Real-time polymerase chain reaction
SYN-Cnr2	CB2R deletion from synapse
Δ^9^-THC	Delta-9-tetrahydrocannabinol
TH	Tyrosine hydroxylase
UTR	Untranslated region
VGLUT2	Vesicular glutamate transporter
VTA	Ventral tegmental area
WT	Wild type
